# Supplementary material for: Genomic Features of Parthenogenetic Animals
Source: J Hered. 2020 Sep 28;112(1):19–33. doi: 10.1093/jhered/esaa031 (PMC7953838; doi:10.1093/jhered/esaa031)
Supplement: esaa031_suppl_Supplementary_Table_1 [file esaa031_suppl_supplementary_table_1.pdf]

Supplementary table 1: Overview of analysed species. This information was collected directly from the cited literature. References include information regarding cellular mode of reproduction, origin of asexuality and/or the age of asexuality.

species	common name	NCBI accesion	cellular mechanism of parthenogenesis	evidence	hybrid origin	age of asexuality [y]	references
Poecilia formosa	amazon molly	SAMN01797685	sperm-dependent functional mitotic	cytology, genetics	yes	100 k	[1]
Adineta vaga	bdelloid rotifer	SAMEA2043852	functional mitotic	stained karyotypes have no apparent chromosome pairs		46 M	[2, 3]
Adineta ricciae	bdelloid rotifer	SAMEA104393659	functional mitotic			46 M	[3, 4]
Rotaria macrura	bdelloid rotifer	SAMEA104393678	functional mitotic	no direct evidence[1]		46 M	[5, 6]
Rotaria magnacalcarata	bdelloid rotifer	SAMEA104393684	functional mitotic	no direct evidence[1]		46 M	[5, 6]
Leptopilina clavipes	parasitoid wasp	SAMN02047179	gamete duplication[2]	cytology	no	6-43 k	[7]
Trichogramma pretiosum	trichogramma wasp	SAMN02439301	gamete duplication[2]	cytology, genetic markers	no	"few"	[8, 9]
Ooceraea biroi[3]	raider ant	SAMN02428046	central fusion	cytology, RAD seq	no		[10]
Apis mellifera capensis	cape honey bee	SAMN10245904 SAMN10245906	central fusion	cytology	no	20	[11]
Aptinothrips rufus	thrip		gamete duplication[4]		no	150-200 k	[12]
Folsomia candida	springtail	SAMN04196550	terminal fusion[4]	cytology	no		[13]
Daphnia pulex	water flea	SAMN03964753 SAMN03964750	central fusion equivalent	No separation at meiosis I; abortive meiosis	yes	1-170 k	[14]
Procambarus virginalis	marbled crayfish	SAMN07142640	functional mitotic	microsat study, histological evidence		less than 30	[15–17]
Plectus sambesii	nemotode	SAMN07227113	unknown meiotic	2 meiotic divisions, 2 polar bodies, but no fusions were observed, putative endoduplication			[18]
Mesorhabditis belari	nemotode	SAMEA5150020	unknown meiotic	cytology; 2 meiotic divisions observed			[19]
Diploscapter coronatus	nemotode	SAMD00025087	unknown		yes		[18, 20]

[1] Meiosis was not observed in two bdelloid rotifers *Habrotrocha tridens* and *Philodina roseola*, both members of *Philodinidae*, the same family as *Rotaria*.
[2] *Wolbachia* induced
[3] formerly *Cerapachys biroi*
[4] suggested that it is endosymbiont induced

Diploscapter pachys	nematode	SAMN03456257	functional mitotic	formally central fusion (meiosis I skipped), no recombination, only sister chromatid separation	yes	18 M[5]	[21]
Panagrolaimus davidi	nemotode	SAMN02741088	unknown	polar body produced	yes	1.3-8.5 M	[22]
Acrobeloides nanus	nemotode	SAMN06041019	unknown meiotic				[18]
Meloidogyne incognita	root-knot nematode	SAMEA104032784 SAMN05712521	functional mitotic		yes	"recently"	[23–25]
Meloidogyne javanica	root-knot nematode	SAMEA3298191 SAMN05712519	functional mitotic		yes	"recently"	[25]
Meloidogyne arenaria	root-knot nematode	SAMEA3298190 SAMN05712513 SAMN08721831	functional mitotic		yes	"recently"	[25]
Meloidogyne floridensis	peach root-knot nematode	SAMN05712529	unknown	meiotic mechanism suggested by cytology; however the study conflicts in ploidy with the data in this study (see S1)	yes	"recently"	[25, 26]
Meloidogyne enterolobii	root-knot nematode	SAMN05712528	functional mitotic		yes	"recently"	[25]
Hypsibius dujardini	tardigrade; water bear	SAMEA3679301	terminal fusion equivalent	meiosis II suppressed			[27]
Ramazzottius varieornatus	tardigrade; water bear; Kumamushi	SAMD00054187		no males have been found			[6]

[5] Assuming non-hybrid origin suggested by Hiraki at al. 2017.
[6] personal communication with Mark Blaxter

References

[1] Lamatsch DK, Steinlein C, Schmid M, Schartl M. Noninvasive determination of genome size and ploidy level in fishes by flow cytometry: detection of triploid Poecilia formosa. Cytometry. 2000 feb;39(2):91–95. Available from: https://doi.org/10.1002/(sici)1097-0320(20000201)39:2<91::aid-cyto1>3.0.co;2-4.

[2] Mark Welch JL, Meselson M. Karyotypes of bdelloid rotifers from three families. Hydrobiologia. 1998;387/387:403–407. Available from: https://doi.org/10.1023/a:1017007131386.

[3] Mark Welch DB, Meselson M. Oocyte nuclear DNA content and GC proportion in rotifers of the anciently asexual Class Bdelloidea. Biological Journal of the Linnean Society. 2003 may;79(1):85–91. Available from: https://doi.org/10.1046/j.1095-8312.2003.00180.x.

[4] Pouchkina-Stantcheva NN, McGee BM, Boschetti C, Tolleter D, Chakrabortee S, Popova AV, et al. Functional Divergence of Former Alleles in an Ancient Asexual Invertebrate. Science. 2007 oct;318(5848):268–271. Available from: https://doi.org/10.1126/science.1144363.

[5] Hsu WS. Oogenesis in the Bdelloidea rotifer Philodina roseola Ehrenberg. Cellule. 1956;57:283–296.

[6] Hsu WS. Oogenesis in Habrotrocha tridens (Milne). The Biological Bulletin. 1956;111(3):364–374.

[7] Pannebakker BA, Pijnacker LP, Zwaan BJ, Beukeboom LW. Cytology of Wolbachia-induced parthenogenesis in Leptopilina clavipes (Hymenoptera: Figitidae). Genome. 2004 apr;47(2):299–303. Available from: https://doi.org/10.1139/g03-137.

[8] Ardila-Garcia AM, Umphrey GJ, Gregory TR. An expansion of the genome size dataset for the insect order Hymenoptera, with a first test of parasitism and eusociality as possible constraints. Insect Molecular Biology. 2010 feb;19(3):337–346. Available from: https://doi.org/10.1111/j.1365-2583.2010.00992.x.

[9] Gokhman VE, Pereira FF, Costa MA. A cytogenetic study of three parasitic wasp species (Hymenoptera, Chalcidoidea, Eulophidae, Trichogrammatidae) from Brazil using chromosome morphometrics and base-specific fluorochrome staining. Comparative Cytogenetics. 2017 mar;11(1):179–188. Available from: https://doi.org/10.3897/compcytogen.v11i1.11706.

[10] Oxley PR, Ji L, Fetter-Pruneda I, McKenzie SK, Li C, Hu H, et al. The Genome of the Clonal Raider Ant Cerapachys biroi. Current Biology. 2014 feb;24(4):451–458. Available from: https://doi.org/10.1016/j.cub.2014.01.018.

[11] Verma S, Ruttner F. Cytological analysis of the thelytokous parthenogenesis in the Cape honeybee (Apis mellifera capensis Escholtz). Apidologie. 1983;14(1):41–57.

[12] Fontcuberta García-Cuenca A, Dumas Z, Schwander T. Extreme genetic diversity in asexual grass thrips populations. Journal of evolutionary biology. 2016;29(5):887–899.

[13] Riparbelli MG, Giordano R, Callaini G. Centrosome inheritance in the parthenogenetic egg of the collembolan Folsomia candida. Cell and Tissue Research. 2006 aug;326(3):861–872. Available from: https://doi.org/10.1007/s00441-006-0253-x.

[14] Hiruta C, Nishida C, Tochinai S. Abortive meiosis in the oogenesis of parthenogenetic Daphnia pulex. Chromosome Research. 2010;18(7):833–840.

[15] Vogt G, Tolley L, Scholtz G. Life stages and reproductive components of the Marmorkrebs (marbled crayfish), the first parthenogenetic decapod crustacean. Journal of Morphology. 2004;261(3):286–311. Available from: https://doi.org/10.1002/jmor.10250.

[16] Martin P, Thonagel S, Scholtz G. The parthenogenetic Marmorkrebs (Malacostraca: Decapoda: Cambaridae) is a triploid organism. Journal of Zoological Systematics and Evolutionary Research. 2015 nov;54(1):13–21. Available from: https://doi.org/10.1111/jzs.12114.

[17] Vogt G, Falckenhayn C, Schrimpf A, Schmid K, Hanna K, Panteleit J, et al. The marbled crayfish as a paradigm for saltational speciation by autopolyploidy and parthenogenesis in animals. Biology open. 2015;p. bio–014241.

[18] Lahl V, Sadler B, Schierenberg E. Egg development in parthenogenetic nematodes: variations in meiosis and axis formation. The International Journal of Developmental Biology. 2006;50(4):393–397. Available from: https://doi.org/10.1387/ijdb.052030vl.

[19] Grosmaire M, Launay C, Siegwald M, Brugière T, Estrada-Virrueta L, Berger D, et al. Males as somatic investment in a parthenogenetic nematode. Science. 2019;363(6432):1210–1213.

[20] Hiraki H, Kagoshima H, Kraus C, Schiffer PH, Ueta Y, Kroiher M, et al. Genome analysis of Diploscapter coronatus: insights into molecular peculiarities of a nematode with parthenogenetic reproduction. BMC Genomics. 2017 jun;18(1). Available from: https://doi.org/10.1186/s12864-017-3860-x.

[21] Fradin H, Kiontke K, Zegar C, Gutwein M, Lucas J, Kovtun M, et al. Genome Architecture and Evolution of a Unichromosomal Asexual Nematode. Current Biology. 2017 oct;27(19):2928–2939.e6. Available from: https://doi.org/10.1016/j.cub.2017.08.038.

[22] Schiffer PH, Danchin E, Burnell AM, Schiffer AM, Creevey C, Wong S, et al. Signatures of the evolution of parthenogenesis and cryptobiosis in panagrolaimid nematodes. bioRxiv. 2018;Available from: https://www.biorxiv.org/content/early/2018/06/27/159152.

[23] Triantaphyllou A. Oogenesis and the Chromosomes of the Parthenogenic Root-knot Nematode Meloidogyne incognita. Journal of nematology. 1981;13(2):95.

[24] Van der Beek JG, Los JA, Pijnacker LP. Cytology of parthenogenesis of five Meloidogyne species. Fundamental and applied nematology. 1998;21(4):393–399.

[25] Lunt DH, Kumar S, Koutsovoulos G, Blaxter ML. The complex hybrid origins of the root knot nematodes revealed through comparative genomics. PeerJ. 2014;2:e356.

[26] Handoo Z, Nyczepir A, Esmenjaud D, Van der Beek J, Castagnone-Sereno P, Carta L, et al. Morphological, molecular, and differential-host characterization of Meloidogyne floridensis n. sp.(Nematoda: Meloidogynidae), a root-knot nematode parasitizing peach in Florida. Journal of nematology. 2004;36(1):20.

[27] Ammermann D. Die Cytologie der Parthenogenese bei dem Tardigraden Hypsibius dujardini. Chromosoma. 1967;23(2):203–213. Available from: https://doi.org/10.1007/bf00331113.
